# Diclofenac: A Nonsteroidal Anti-Inflammatory Drug Inducing Cancer Cell Death by Inhibiting Microtubule Polymerization and Autophagy Flux

**DOI:** 10.3390/antiox11051009

**Published:** 2022-05-20

**Authors:** Soohee Choi, Suree Kim, Jiyoung Park, Seung Eun Lee, Chaewon Kim, Dongmin Kang

**Affiliations:** Department of Life Science, Fluorescence Core Imaging Center, Ewha Womans University, Seoul 03760, Korea; mhoiing@naver.com (S.C.); kimsuree@hanmail.net (S.K.); jypark89@ewhain.net (J.P.); sky960425@naver.com (S.E.L.); kcw4269@naver.com (C.K.)

**Keywords:** diclofenac, autophagy, microtubule depolymerization, cell death, combination cancer therapy

## Abstract

Diclofenac, a nonsteroidal anti-inflammatory drug (NSAID) used to treat inflammatory diseases induces cellular toxicity by increasing the production of reactive oxygen species (ROS) and impairing autophagic flux. In this study, we investigated whether diclofenac induces cancer cell death and the mechanism by which diclofenac causes cell death. We observed that diclofenac induces mitotic arrest with a half-maximal effective concentration of 170 μM and cell death with a half-maximal lethal dose of 200 µM during 18-h incubation in HeLa cells. Cellular microtubule imaging and in vitro tubulin polymerization assays demonstrated that treatment with diclofenac elicits microtubule destabilization. Autophagy relies on microtubule-mediated transport and the fusion of autophagic vesicles. We observed that diclofenac inhibits both phagophore movement, an early step of autophagy, and the fusion of autophagosomes and lysosomes, a late step of autophagy. Diclofenac also induces the fragmentation of mitochondria and the Golgi during cell death. We found that diclofenac induces cell death further in combination with 5-fuorouracil, a DNA replication inhibitor than in single treatment in cancer cells. Pancreatic cancer cells, which have high basal autophagy, are particularly sensitive to cell death by diclofenac. Our study suggests that microtubule destabilization by diclofenac induces cancer cell death via compromised spindle assembly checkpoints and increased ROS through impaired autophagy flux. Diclofenac may be a candidate therapeutic drug in certain type of cancers by inhibiting microtubule-mediated cellular events in combination with clinically utilized nucleoside metabolic inhibitors, including 5-fluorouracil, to block cancer cell proliferation.

## 1. Introduction

Non-steroidal anti-inflammatory drugs (NSAIDs) are chemical inhibitors of cyclooxygenase enzyme (COX), conferring pain relief and reducing inflammation. Most NSAIDs inhibit both COX-1 and COX-2 and have severe side effects, such as renal toxicity, stomach irritation, cardiac diseases, and hepatotoxicity [1,2,3,4]. NSAIDs are proposed to increase mitochondrial oxidative stress by inhibiting the mitochondrial electron transport chain complex I and thereby inducing the production of superoxide anions from leaked electrons [5,6]. Increased mitochondrial reactive oxygen species (ROS) elicit apoptotic cell death. Diclofenac, an NSAID, is implicated in hepatotoxicity by impairing autophagic flux and lysosome function [7]. Diclofenac induces the production of mitochondrial ROS and, therefore, increased accumulation of damaged mitochondria followed by mitochondrial dysfunction [6,7]. Considering that rapamycin-induced autophagy ameliorates diclofenac-induced cell death, the inhibition of autophagic flux by diclofenac followed by increased levels of ROS is a major cause of hepatotoxicity. Which cellular target of diclofenac is modified during autophagic flux and the mechanism by which diclofenac affects the autophagy process have not been determined.

Macroautophagy (hereafter referred to as autophagy) is a critical physiological event in relieving oxidative stress and protecting cells from ROS-induced death. Increased intracellular ROS result in autophagy initiation by inhibiting mTORC1 activity, disrupting the association of Beclin-1 with Bcl-2, which is a negative regulator of Beclin-1, and increasing the expression of autophagy effectors such as LC3, p62, and autophagy-related gene 5 (ATG5) [8,9,10]. This autophagic induction is important in reducing oxidative stress by removing damaged mitochondria and other malfunctioned organelles and to maintain intracellular redox balance. Autophagy is actively implicated in cell survival based on the observations that in vivo knockout of *Atg5* [11] or *Atg7* [12] in the central nervous system of mice causes neuronal cell death and the deletion of Beclin-1 in *Caenorhabditis elegans* triggers programmed cell death [13]. These studies support the concept that autophagy is required for cell survival. Several works have shown that types of cell death vary depending on which stages of autophagy are inhibited. The inhibition of early autophagy by genetic deletion of autophagy-related genes such as *Beclin-1*, *Atg5*, or *VPS34* induces apoptosis without autophagic vacuoles [14]. However, the blocking of late autophagy by genetic depletion of the LAMP2 lysosomal protein or the administration of lysosomal inhibitors (such as chloroquine and bafilomycin A, an inhibitor of vacuolar ATPase into cells) causes autophagic cell death with vacuoles [14,15]. In nutrient-starved cells, the inhibition of autophagy results in an accelerated apoptosis following caspase activation [15]. Prolonged inhibition of mitophagy (a form of autophagy that selectively degrades damaged mitochondria) is thought to induce an increase in mitochondrial ROS levels. The resulting cytosolic hydrogen peroxide (H_2_O_2_) can inhibit autophagy further and finally achieve irreversible apoptotic cell death. A recent study revealed the H_2_O_2_-dependent inhibition of early autophagy in aged cells. ATG3 and ATG7 are two critical effectors for autophagosome formation. Thiol residue oxidation of ATG3 and ATG7 by exogenous H_2_O_2_ molecules or endogenous H_2_O_2_ molecules in aged mouse aorta inhibits the conjugation of LC3 to phosphatidylethanolamine and autophagy progression such as autophagosome formation, maturation, and autophagosome–lysosome fusion [16]. The proposal that the mutual inhibition between autophagy and apoptosis exists to some degree [17] is supported by redox homeostasis in the cell. In cells under mild oxidative stress, autophagy is activated to reduce ROS levels and protect cells from apoptosis. On the other hand, in cells under high oxidative stress, autophagy is inhibited through the inactivation of autophagy-related proteins by H_2_O_2_ molecules followed by the accumulation of damaged mitochondria and cell death, including apoptosis. H_2_O_2_ acts as a promoter for growth and proliferation and many cancer cells have a high level of H_2_O_2_ compared to normal cells [18]. Some cancer cells have high autophagy activity and require autophagy for growth and maintenance [19]. Based on the previous findings, a candidate agent that both inhibits autophagy and compromises redox balance will be a promising therapeutic drug for certain types of cancer.

Experimental, epidemiologic, and clinical studies have reported that the use of NSAIDs is associated with the prevention of tumorigenesis and the inhibition of cancer progression [20,21,22]. A variety of NSAIDs show inhibitory effects on tumor cell growth but their cell death-inducing mechanism does not seem to be associated with COX inhibition because the growth half-maximal inhibitory concentration (IC_50_) of tested NSAIDs is 58–400 times as high as COX-1 IC_50_ or COX-2 IC_50_ in cultured cancer cells [20]. With regard to cell toxicity, diclofenac is suggested to suppress autophagy progression through ROS production and induced lysosomal defect [7]. Depending on the cancer cell type and stage, autophagy not only acts as a suppressor of tumorigenesis, but also works as a promoter of tumor growth and resistance to chemotherapies [19,23,24,25].

Here, we have examined whether diclofenac affects cancer cell cycle progression and the mode of action of diclofenac on cytotoxicity to cancer cells. We found that diclofenac inhibits mitotic progression by destabilizing microtubules in HeLa and HepG2 cells. The inhibition of microtubule polymerization by diclofenac results in compromised autophagic flux and the fragmentation of mitochondria and the Golgi apparatus, indicating apoptosis. We observed that the combination of diclofenac with 5-fluorouracil, a clinically used DNA replication-inhibiting anticancer drug, confers a synergistic anti-proliferative effect.

## 2. Materials and Methods

### 2.1. Materials

Diclofenac sodium (D6899), paclitaxel (T7402) (taxol), and nocodazole were from Sigma Aldrich (St. Louis, MO, USA). For flow cytometry, RNase and propidium iodide were obtained from Sigma Aldrich. Rapamycin (TLRL-rap) and normal horse serum were from Invitrogen (Carlsbad, CA, USA). Rotenone and bafilomycin A1 (B1793) were purchased from Sigma Aldrich (St Louis, MO, USA). SAR405 (HY-12481), a Vps34 inhibitor, was from Medchem Express (Monmouth Junction, NJ, USA). MitoSOX™ Red mitochondrial superoxide indicator and Hochest 33342 were from Invitrogen (Carlsbad, CA, USA). Adenovirus expressing mCherry-GFP-LC3 was obtained from Byung-Hoon Lee (Seoul National University, Seoul, South Korea). HeLa cells stably expressing histone H2B–GFP were obtained from J.H. Lee (Ajou University, Suwon, South Korea). Mouse monoclonal antibodies to α-tubulin (A11126) were obtained from Sigma Aldrich (St. Louis, MO, USA). The plasmids for mammalian cell expression of mCherry-WDFY (WD repeat and FYVE domain-containing) and GFP-LC3 were obtained from Won Do Heo (KAIST, Daejeon, South Korea) and from Hyung Jin Son (Ewha Womans University, Seoul, South Korea), respectively. Rabbit polyclonal antibodies to LC3 (L8918) and mouse monoclonal antibodies to β-actin (A2228) were purchased from Sigma Aldrich (St Louis, MO, USA). Mouse monoclonal antibodies to p62 (ab56416) were from Abcam (Cambridge, UK). Rabbit monoclonal antibodies to mTOR (2983) and to LAMP1 (9091) were purchased from Cell Signaling Technology (Beverly, MA, USA). Mouse monoclonal antibodies to Golgin-97 (A-21270) were from Thermo Fisher Scientific (Waltham, MA, USA). Alexa Fluor 488–conjugated secondary antibodies to rabbit IgG (A11034), Alexa Fluor 546–conjugated secondary antibodies to mouse IgG (A11030), Alexa Fluor 647–conjugated secondary antibodies to mouse IgG, and Alexa Fluor 546-conjugated secondary antibodies to rabbit IgG (A11035) were purchased from Invitrogen (Carlsbad, CA, USA). 4–6-Diamidino-2-phenylindole (DAPI) was from Roche (Basel, Switzerland) and mounting solution Fluoromount-G (0100-01) was from Southern Biotech (Birmingham, AL, USA). The EZ-cytox cell viability assay kit was from Daeilbiotech (Suwon, South Korea). For cell transfection, the Neon Transfection system kit (MPK10096) was from Invitrogen (Carlsbad, CA, USA).

### 2.2. Cell Culture and Transfection

HeLa, HepG2, AsPC-1, and MIA PaCa-2 cells were obtained from ATCC (Manassas, VA, USA) and cultured in high-glucose Dulbecco’s modified eagle medium (LM001-05, Welgene, Gyeongsan, South Korea) containing 10% (*v*/*v*) fetal bovine serum (Gibco, Carlsbad, CA, USA) and 1% penicillin/streptomycin (Hyclone, Waltham, MA, USA). For starvation to induce autophagy, cells were cultured in Earle’s balanced salt solution (EBSS; CA006-050, GenDEPOT, Katy, TX, USA). All cells were grown at 37 °C in a humidified atmosphere of 5% CO_2_ incubator. For infection of mCherry-GFP-LC3 adenovirus, HepG2 cells were seeded in 12-well plates and incubated with the adenovirus the following day for 16 h. For transfection of mCherry-WDFY and GFP-LC3 into HepG2 cells, Neon electroporation (Invitrogen, Carlsbad, CA, USA) was performed according to manufacturer’s directions.

### 2.3. Immunofluorescence and Confocal Microscopy

Immunofluorescence analysis was performed as described previously, with some modifications [26]. Cells were cultured in 12-well dishes containing cover slips (diameter, 18 mm) coated with poly-L-lysine for immunofluorescence staining. To stain β-tubulin of LAMP1 (lysosome marker), cells were fixed for 10 min in 100% methanol or 4% formaldehyde in phosphate-buffered saline (PBS). The cells were incubated with blocking solution (5% normal horse serum, 0.1% Triton X-100 in PBS) for 30 min. The cells then were incubated with primary antibodies in blocking solution for 30 min at room temperature (anti-LAMP1, 1:200 dilution; anti-Golgin97, 1:500). After washing three times with PBS, cells were incubated with Alexa Fluor-conjugated secondary antibodies at a 1:1000 dilution in blocking solution. To stain DNA, DAPI, (0.2 μg/mL) was used. Samples were mounted onto slide glasses using Fluoromount-G (Southern Biotech, Birmingham, AL, USA). The fluorescence images were obtained using a confocal microscope (LSM 880 Airy, Carl Zeiss, Göttingen, Germany) located at Fluorescence Core Imaging Center (Ewha Womans University, Seoul, South Korea). To image live HeLa cells stably expressing histone H2B–GFP, cells were cultured in 12-well dishes containing cover slips (diameter, 18 mm). Confocal images were acquired using an LSM 880 Airy microscope equipped with an incubation chamber in an atmosphere of 5% CO_2_ to measure chromosomal condensation and cell rounding and then analyzed with Zen software (Carl Zeiss, Göttingen, Germany) or NIS Elements software 3.1 (Nikon, Tokyo, Japan).

### 2.4. Quantitative Analysis of Images

To measure autophagic flux, autophagosomes were counted as yellow spots (mCherry^+^/GFP^+^) and autolysosomes were counted as red spots (mCherry^+^/GFP^−^) in cells expressing mCherry-GFP as described previously [27]. A colocalization module was used to measure overlapping spots. For measuring mean intensity, three or four z-stack images were merged and analyzed by NIS Elements software 3.1.

### 2.5. Flow Cytometry

Flow cytometry was conducted with a FACS Calibur flow cytometer (BD sciences, San Jose, CA, USA). For analysis of cell cycle stage, cells (5 × 10^5^/mL) were washed twice with ice-cold PBS, fixed overnight at 4 °C in 70% ethanol, and stained with 1 mL of a solution containing RNase (50 μg/mL) and propidium iodide (50 μg/mL) for 30 min at 37 °C without light. For data analysis, FlowJo 7.6 (BD sciences, San Jose, CA, USA) was used to estimate cell cycle phases.

### 2.6. Cell Viability Assay

Cell viability was measured using EZ-cytox water-soluble tetrazolium salt (Daeilbiotech, Suwon, Korea) according to the manufacturer’s manual. Cells (10^4^ cells per well of a 96-well plate) were seeded and incubated for 14 h. Cells were treated with various concentrations of tested chemicals for 18 h. A 10-μL aliquot of a detection reagent in the kit was added into each well (100 μL) and incubated for 1 h for 37 °C The absorbance at 450 nm was measured using a SpectraMax M Series Multi-Mode Microplate Readers (Molecular Devices, San Jose, CA, USA) at the Fluorescence Core Imaging Center.

### 2.7. Microtubule Polymerization Assessment

A microtubule assay was performed to investigate the effects of diclofenac on microtubule polymerization. HeLa cells were seeded in 12-well dishes containing cover slips (diameter, 18 mm) coated with poly-L-lysine. Cells were incubated in a medium containing tested chemicals at 37 °C for 6 h or 37 °C for 3 h and placed on ice for 3 h. Cells were fixed and subjected to immunofluorescence staining in accordance with Section 2.3 using antibodies to α-tubulin (1:10,000 dilution).

### 2.8. In Vitro Tubulin Polymerization Assay

To investigate whether diclofenac affects microtubule polymerization directly, an in-vitro tubulin polymerization assay was performed using the tubulin polymerization assay kit (BK006P; Cytoskeleton, Denver, CO, USA) according to the manufacturer’s instructions. The mixtures contained tubulin in the presence of 0.01% dimethyl sulfoxide (DMSO) and one of the following: 10 µM taxol, 10 µM nocodazole, or diclofenac (0.17 mM or 1.7 mM). These mixtures were incubated at 37 °C for 30 min. The absorbance at 340 nm was measured to monitor tubulin polymerization using SpectraMax M Series Multi-Mode Microplate Readers (Molecular Devices, San Jose, CA, USA).

### 2.9. Immunoblot Analysis

Cells were harvested and sonicated with cold lysis buffer (20 mM Tris-HCl, pH 7.5, 0.15 M NaCl, 5% glycerol, 0.1% NP-40, 1 mM Na_3_VO_4_, 5 mM NaF, 10 μg/mL aprotinin, 10 μg/mL leupeptin, 1 mM phenylmethylsulfonyl fluoride, 1 mM DTT, and a phosphatase inhibitor cocktail [Sigma Aldrich, St. Louis, MO, USA]). The homogenates were centrifuged at 12,000× *g*, 4 °C for 15 min. The resulting supernatants were used for immunoblot analyses. Samples were loaded onto a sodium dodecyl sulfate-polyacrylamide gel electrophoresis gel and separated by electrophoresis. The proteins were transferred onto an activated polyvinylidene difluoride membrane with 0.45 μm pore size (Millipore, Darmstadt, Germany) using methanol with transfer buffer (3.03 g/L Tris, 14.17 g/L glycine, 20% methanol). The membrane was incubated with 5% bovine serum albumin in tween-20 Tris-buffered saline at room temperature for 20 min using a rocker, followed by incubation at 4 °C overnight on a rocker with antibodies (1:2000 dilution). Immune complexes were detected with horseradish peroxidase conjugated secondary antibodies (Bio-Rad, Hercules, CA, USA) and enhanced with chemiluminescence reagents (Ab Frontier, Daejeon, Korea) using the IQ800 imaging system (GE Healthcare, Sweden). The abundance of target proteins was quantified by densitometric analysis of immunoblots. To measure protein concentrations, Bradford assay (Bio-Rad) data were acquired using a SpectraMax M2 Microplate Reader (Molecular Devices, San Jose, CA, USA) at the Fluorescence Core Imaging Center.

### 2.10. Mitochondrial Network Analysis

HepG2 cells were cultured in 12-well dishes containing cover slips (diameter, 18 mm,) coated with poly-L-lysine. Cells were treated with tested chemicals. For co-treatment of *n*-acetylcysteine and diclofenac, *n*-acetylcysteine was added into cells 1 h prior to diclofenac treatment. To stain mitochondria, cells were incubated with 250 nM of Mitotracker (M7512; Invitrogen, Carlsbad, CA, USA) for 30 min at 37 °C. Cells were fixed with 4% formaldehyde, washed with PBS, and observed using a confocal microscope (LSM 880; Carl Zeiss, Göttingen, Germany). Quantitative analysis for mean rod/branch length and median rod/branch length was performed to measure the fragmented mitochondrial network using the Mitochondrial Network Analysis (MiNA) toolset applied with NIH Image J (Bethesda, MD, USA) as described previously [28].

### 2.11. MitoSOX Imaging

HepG2 cells were cultured in a 96-well plate at a density of 400,000 cells. The next day, cells were incubated with a medium containing 1 μM MitoSOX for 10 min at 37 °C. Cells were then washed with PBS three times and treated with test chemicals for 8 h. For co-treatment of *n*-acetylcysteine and diclofenac, *n*-acetylcysteine was added to cells 1 h prior to diclofenac treatment. Cells were washed with PBS three times and then incubated with a medium containing 3 μM Hoechst 33342 for 10 min to visualize nuclear DNA. Live cells were observed using an ImageXpress Confocal HT.ai (Molecular Devices, San Jose, CA, USA) at the Fluorescence Core Imaging Center. Quantitative analysis was performed to measure the mean value of red fluorescence intensity per cell with MetaXpress 6 software (Molecular Devices, San Jose, CA, USA). A cell boundary was determined using the nucleus signal with Hochest 33342 and a custom module.

### 2.12. Statistical Analysis

All quantitative data are presented as means ± SD from multiple experiments. Data were analyzed using Student’s t-test on Sigma Plot 10.0 software (Inpixon, Palo Alto, CA, USA). A *p*-value < 0.05 was considered statistically significant.

## 3. Results

### 3.1. Diclofenac Inhibits Mitotic Progression in HeLa Cells

**Figure 1:**
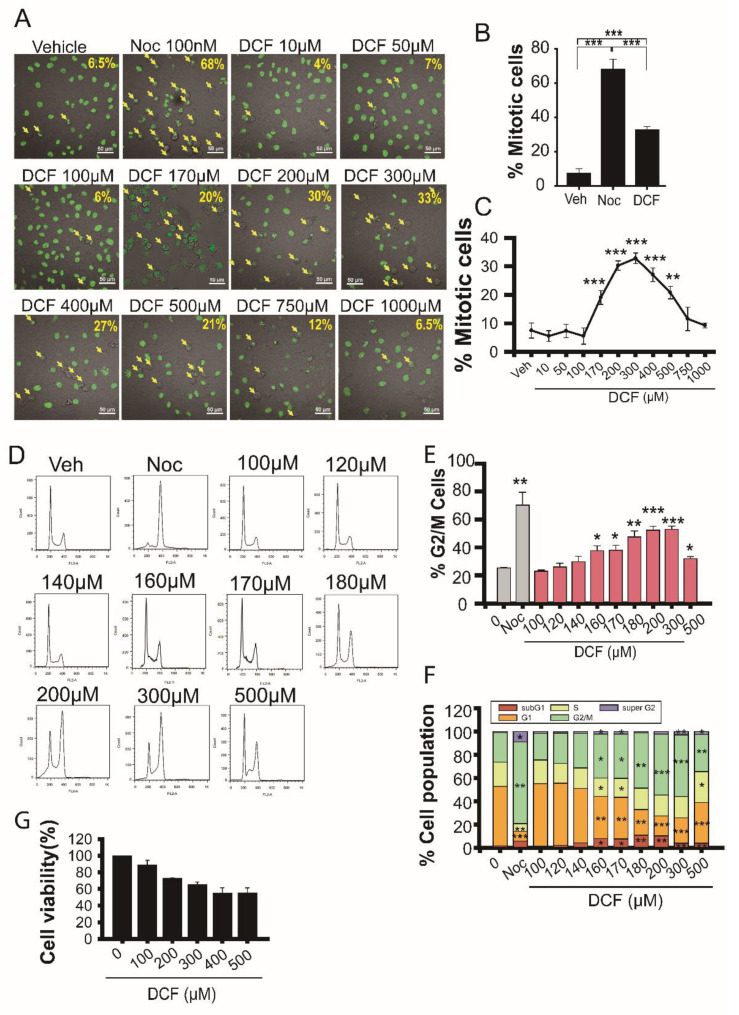
Diclofenac inhibits mitotic progression in HeLa cells. (**A**) HeLa cells expressing human histone H2B conjugated with green fluorescent protein were incubated in a medium containing vehicle (0.1% dimethyl sulfoxide [DMSO]), nocodazole (Noc, 100 nM), or various concentrations of diclofenac (DCF) for 18 h and then observed using a Nikon A1R confocal microscope. Scale bar, 50 µm. The arrows show mitotic cells. (**B**,**C**) The percentage of mitotic cells was evaluated on the basis of chromosome condensation. Data are presented as means ± SD from three independent experiments (*n* = 186–337 cells). Cells were treated with 100 nM of nocodazole (Noc) or 300 µM of diclofenac (DCF). (**D**–**F**) HeLa cells were incubated in a medium containing vehicle (0.1% DMSO), nocodazole (Noc, 100 nM) or various concentrations of diclofenac (DCF) for 18 h. Cell cycle stages were determined by flow cytometry. Cells containing 2n or 4n DNA are shown in histograms in part (**D**). The percentage of cells at G_2_ and mitosis (G_2_/M) (**E**) or at each stage (**F**) is displayed on the base of the histogram. Diclofenac causes mitotic arrest. Data are presented as means ± SD from three independent experiments. (**G**) Cell viability assay using a water-soluble tetrazolium salt (EZ-cytox) shows that diclofenac with a half-maximal lethal dose of 200 µM has a cytotoxic effect on HeLa cells. Data are presented as means ± SD from three independent experiments. * *p* < 0.05; ** *p* < 0.01; *** *p* < 0.001 (Student’s *t*-test).

Diclofenac is the most potent autophagy inhibitor among five tested NSAIDs (including aceclofenac, etodolac, sulindac, and ketorolac) and induces hepatotoxicity via harmful ROS production [7]. High levels of H_2_O_2_ induce cell cycle arrest at the G_0_ and G_1_ phases [29,30]. To examine the effect of diclofenac on cell growth and proliferation, we tested whether diclofenac induces cytotoxicity via cell cycle arrest. HeLa cells stably expressing a human histone H2B conjugated with green fluorescent protein (H2B-GFP) were used to monitor mitotic progression by estimating chromosome condensation. Early mitotic cells were scored on the basis of chromosome condensation in rounded cells (Figure 1A–C). The number of mitotic cells increased by 5 times in cells treated with 300 μM of diclofenac for 18 h compared with untreated cells. GFP signal represents the nucleus in H2B-GFP HeLa cells. We carried out flow cytometry experiments to monitor cell cycle arrest in HeLa cells (Figure 1D–F). Diclofenac induced mitotic arrest until 300 μM was reached (Figure 1C), and the percentage of mitotic cells decreased at >300 μM diclofenac (Figure 1F). Prolonged incubation with higher diclofenac concentrations caused mitotic cell death through a mitotic catastrophe because of deranged mitotic spindle formation [31]; consequently, the cells on the culture plate became detached. Figure 1F shows that the pool of subG_1_ cells increased slightly at concentrations above 140 μM diclofenac compared to untreated control cells (Figure 1F), indicating that a small portion of the cell death pool was captured by flow cytometry analysis.

The mitotic arrest effect of diclofenac (300 µM) was as low as that of nocodazole (100 nM) by 50% in HeLa cells. Cell viability assays using tetrazolium salt revealed that diclofenac treatment for 18 h induces cell death in a concentration-dependent manner up to a concentration of 400 μM (Figure 1G). Extensive chromosome instability of HeLa cells may cause resistance to cell death [32] induced by treatment with high concentrations of diclofenac. Diclofenac results in G_2_/M arrest with a half-maximal effective concentration of 170 μM and a death-inducing effect with a half-maximal lethal dose of 200 µM in HeLa cells.

### 3.2. Diclofenac Induces Microtubule Depolymerization

**Figure 2:**
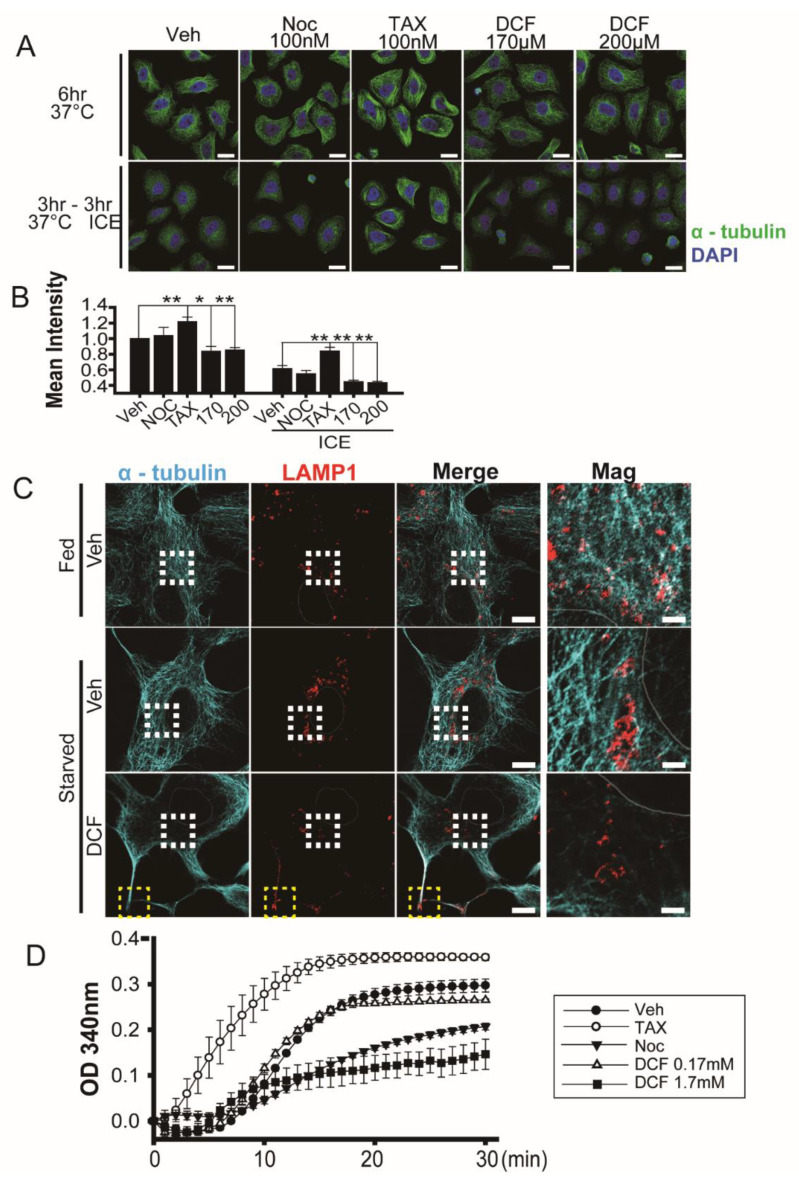
Diclofenac induces microtubule depolymerization. (**A**) Confocal microscopy of HeLa cells stained with antibodies to α-tubulin (green) after incubation at 37 °C (upper panel) for 6 h or at 37 °C for 3 h followed by 4 °C for 3 h. Cells were incubated in a medium containing vehicle (0.1% dimethyl sulfoxide [DMSO]), nocodazole (100 nM), taxol (100 nM), or diclofenac (170 µM, 200 µM). Nuclei were stained with 4–6-diamidino-2-phenylindole (DAPI, blue). (**B**) Quantitative analysis of mean fluorescence intensity of α-tubulin. Data are presented as means ± SD from three independent experiments (*n* = 52–60 cells). * *p* < 0.05; ** *p* < 0.01 (Student’s *t*-test). (**C**) Confocal microscopy of HepG2 cells stained with antibodies to α-tubulin (green) and LAMP1 (red) after incubation in a medium containing vehicle (0.1% DMSO) or diclofenac (500 µM) under fed (Dulbecco’s modified eagle medium, 10% fetal bovine serum) conditions or starved (Earle’s balanced salt solution) conditions for 8 h. Nuclei were stained with DAPI (blue). Representative images are shown. (*n* = 14–18 cell). Areas enclosed by the white boxes are shown at higher magnification. Yellow boxes indicate the edges of the plasma membrane. Three independent experiments were performed. Scale bar, 10 µm; scale bar in magnification; 2 µm. (**D**) In vitro tubulin polymerization. Polymerization activity was monitored in the presence of DMSO (0.01%, vehicle), taxol (10 μM), nocodazole (10 μM), or diclofenac (0.17 mM and 1.7 mM) for 30 min at 37 °C as the increase in A_340_ nm. Two independent experiments were performed.

Both nocodazole, a microtubule destabilizing drug, and taxol, a microtubule stabilizer, induce mitotic arrest at the metaphase or metaphase/anaphase boundary by changing spindle microtubule dynamics [33,34]. To test for the same effect in diclofenac, we performed cold-induced microtubule depolymerization experiments [35]. Fluorescence intensity of α-tubulin in HeLa cells incubated at 37 °C for 6 h or at 37 °C for 3 h followed by 4 °C for 3 h indicates that diclofenac induces microtubule depolymerization (Figure 2A). Depolymerization activity of diclofenac at 170 μM and 200 μM is greater than that of nocodazole at 100 nM. As expected, taxol (100 nM) induces microtubule polymerization (see Figure 2A). Thus, we observe that diclofenac weakens the overall microtubule network in HeLa cells. Figure 2B offers a graphical display of relative fluorescence intensity of α-tubulin from Figure 2A.

Immunofluorescence experiments indicate that diclofenac also induced microtubule depolymerization and affected localization of lysosomes in HepG2 cells (Figure 2C). In an EBSS (nutrient-starved) medium, lysosomes and autophagosomes are relocated in the perinuclear area to resolve increased intracellular pH [36]. Although lysosomes were distributed well into the cytosol in the presence of nutrients (Fed, Figure 2C top row), perinuclear clustering of lysosomes appeared in the absence of nutrients (Starved, Figure 2C middle row) as reported in previous studies [36,37]. However, diclofenac treatment resulted in the abnormal location of lysosomes near the edges of the plasma membrane in cells (Figure 2C bottom row, yellow box) and reduced numbers of perinuclear lysosomes (Figure 2C bottom row, white box).

To examine whether diclofenac interferes with microtubule polymerization directly, we performed an in vitro assay to monitor tubulin polymerization. Figure 2D shows that 1.7 mM diclofenac (a concentration 10 times higher than that for mitotic arrest in HeLa cells) inhibits tubulin polymerization more effectively than does 10 µM nocodazole (a concentration 100 times higher than that for mitotic arrest in HeLa cells). The effects of diclofenac on microtubule depolymerization were observed in HeLa and HepG2 cells.

### 3.3. Diclofenac Inhibits Autophagy Flux

**Figure 3:**
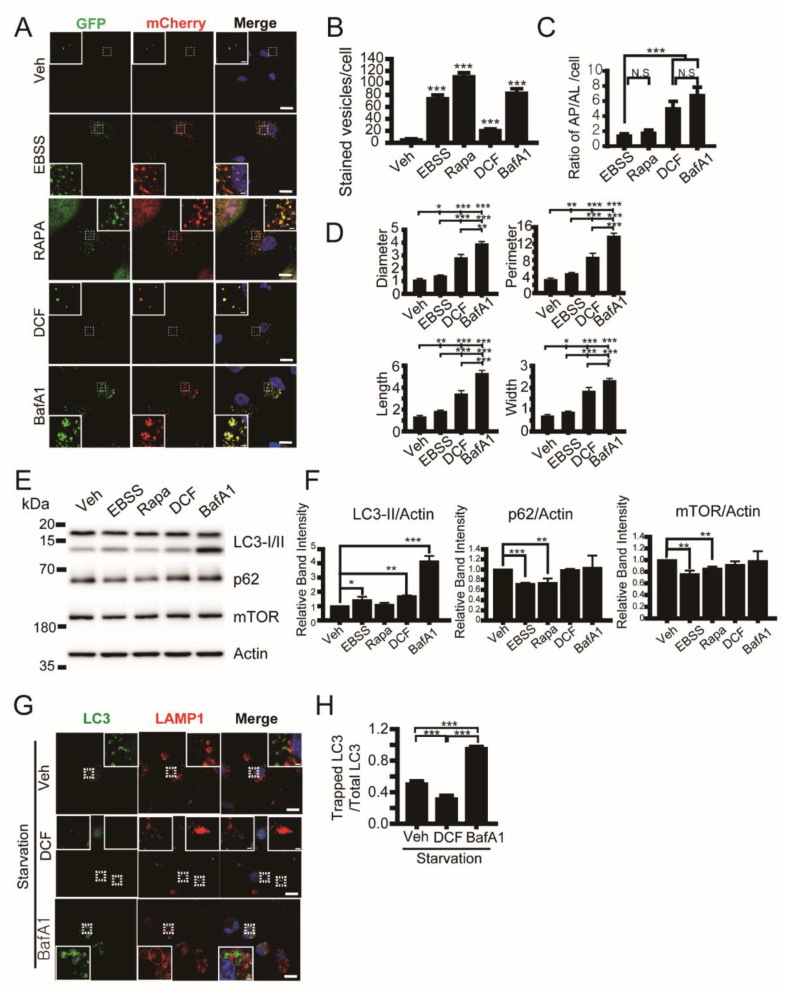
Diclofenac inhibits the fusion of autophagosomes and lysosomes. (**A**) Confocal microscopy of HepG2 cells expressing mCherry-GFP-LC3 were incubated in a medium containing vehicle (0.1% dimethyl sulfoxide [DMSO]), Earle’s balanced salt solution (EBSS), rapamycin (0.25 µM), diclofenac (500 µM), or bafilomycin A1 (100 nM) for 8 h. Autophagosomes appear as yellow spots (mCherry^+^/GFP^+^) and autolysosomes appear as red spots (mCherry^+^/GFP^−^) in merged images. Nuclei were stained with 4–6-diamidino-2-phenylindole (DAPI, blue). (**B**) Quantification of autophagic vesicle number as the sum of autophagosome (mCherry^+^/GFP^+^, yellow) spot and autolysosome (mCherry^+^/GFP^−^, red) spot number per cell. (**C**) Ratio of number of autophagosomes (AP) to autolysosomes (AL) per cell. (**D**) Size of autophagosomes and autolysosomes was quantified by diameter, perimeter, length, and width of each spot. Data are presented as means ± SD from three independent experiments (*n* = 15 cells). (**E**) HepG2 cells were incubated in a medium containing vehicle (0.1% DMSO), EBSS, rapamycin (0.25 µM), diclofenac (500 µM), or bafilomycin A1 (100 nM) for 8 h. Total cell lysates were subjected to immunoblot analysis with antibodies to the indicated proteins. Uncropped western blots in Figure S1 (**F**) The relative immunoblot intensities of LC3-II, p62, and mTor normalized by those of actin were also determined as means ± SD from three independent experiments. (**G**) Nutrient-starved (EBSS-treated) HepG2 cells expressing GFP-LC3 were incubated in a medium containing vehicle (0.1% DMSO), diclofenac (500 µM), or bafilomycin A1 (100 nM) for 8 h. Cells were then subjected to immunofluorescence analysis with antibodies to LAMP1 (lysosomal marker, red) and observed using confocal microscopy. Nuclei were stained with DAPI. (**H**) Quantification of the ratio of LC3 spots in the lysosome (trapped LC3 spots) to total LC3 spots. Lower values on the Y-axis indicate that a small number of LC3 spots exist in the lysosomes. Data are presented as means ± SD from three independent experiments (*n* = 29–39 cells). * *p* < 0.05; ** *p* < 0.01; *** *p* < 0.001 (Student’s *t*-test). Scale bar, 20 µm; scale bar in inset, 2 µm.

The formation and maturation of autophagic vacuoles depends on the cytoskeleton network, including actin and microtubule polymerization [37,38]. We monitored which stage of autophagy was affected by diclofenac using the adenoviral expression of mCherry-GFP-LC3, which is an efficient tool for tracking autophagic flux [39]. Autophagosomes appear as yellow spots (mCherry^+^/GFP^+^) and autolysosomes appear as red spots (mCherry^+^/GFP^−^), in cells expressing mCherry-GFP-LC3 because GFP is acid-labile and mCherry is acid-stable in the lumen of lysosomes [27,40]. Analysis of the spot number ratio of autolysosomes (mCherry^+^GFP^−^) to autophagosomes (mCherry^+^GFP^+^) is useful for estimating the transition from autophagosome to autolysosome. To assess the autophagic vacuole number, we incubated HepG2 cells in a nutrient-starved (EBSS), medium containing rapamycin (an inducer of autophagy by inhibiting mTorc1 [41]) or bafilomycin A (a vacuolar-type H^+^-ATPase and a blocker of autophagy by inhibiting acidification of the lumen of the lysosome) [42]. We observed that the administration of diclofenac into HepG2 cells increases total autophagic vacuole number compared with control cells (vehicle), and increases the spot number ratio of autophagosomes to autolysosomes more than nutrient-starved cells (EBSS) and rapamycin-induced autophagic cells (Figure 3A–C). This result indicates that diclofenac inhibits the fusion of autophagosomes with lysosomes or increases the pH in the lumen of autolysosomes. An increase in autolysosome size can indicate dysfunction of lysosomes and can lead to accumulation of enlarged autolysosomes [27] as shown in cells treated with bafilomycin A1. In diclofenac-treated cells, autolysosome size is twice as large as that in nutrient-starved cells (Figure 3D), indicating diclofenac may induce an increase in lysosomal pH similar to the result in a previous report [7]. Immunoblot analysis (Figure 3E,F) revealed that diclofenac induced an increase in LC3-II protein level during 8 h of incubation compared with control cells. The levels of p62 and mTOR protein in the lysates from diclofenac-treated cells were the same as those from control cells and bafilomycin A-treated cells. This result indicates that diclofenac works as an autophagy inhibitor like bafilomycin A but not as an autophagy inducer like rapamycin.

To examine the effect of diclofenac on the fusion of autophagosomes and lysosomes, we carried out a colocalization of GFP-LC3 with LAMP1, a lysosomal membrane protein. The relative ratio of lysosome-trapped GFP-LC3 spots to total GFP-LC3 spots was reduced in diclofenac-treated cells by 65% compared with bafilomycin A-treated cells and by 30% compared with control cells under nutrient-starved conditions (Figure 3E,F), indicating that diclofenac inhibits the fusion of autophagosomes and lysosomes strongly. We observed many GFP-LC3 spots in the LAMP1-positive enlarged lysosomes in bafilomycin A-treated cells but very few in diclofenac-treated cells. This suggests that the major inhibitory step of autophagy by diclofenac is the fusion of autophagosomes and lysosomes that is dependent on microtubule polymerization, whereas the major inhibitory step of autophagy by bafilomycin A is lysosomal degradation that requires low luminal pH.

### 3.4. Diclofenac Inhibits Microtubule-Dependent Phagophore Movement at the Early Step of Autophagy

**Figure 4:**
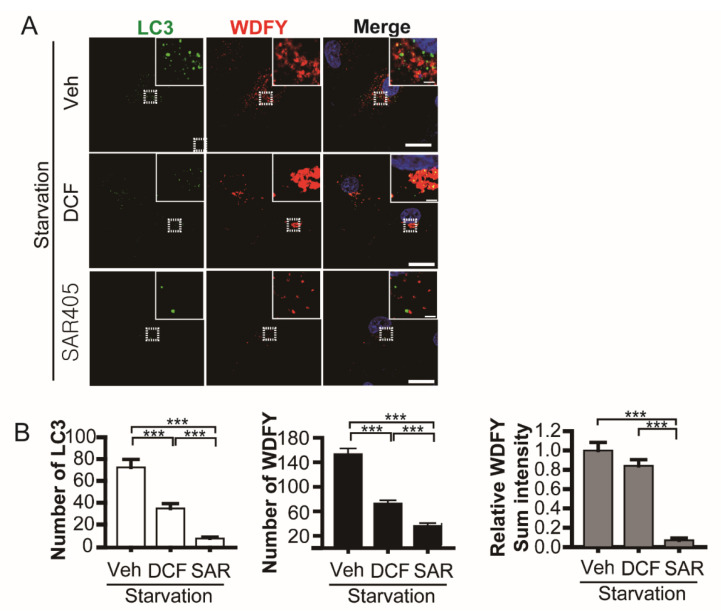
Diclofenac inhibits phagophore movement followed by autophagosome formation. (**A**) Nutrient-starved (Earle’s balanced salt solution) HepG2 cells expressing GFP-LC3 and mCherry-WDFY (PtdIns(3)P reporter) were incubated in a medium containing vehicle (0.1% dimethyl sulfoxide), diclofenac (500 µM), or SAR405 (500 nM) for 8 h. Nuclei were stained with 4–6-diamidino-2-phenylindole. Images were obtained from confocal microscopy. Scale bars, 20 µm; scale bar in insets, 2 µm. (**B**) Quantification of images from (**A**). The number of LC3 spots (left) and WDFY spots (middle) per cell. Relative sum intensity of WDFY fluorescence per cell (right) was measured, which reveals all PtdIns(3)P signals including aggregates as shown in diclofenac-treated cells. Data are presented as means ± SD from three independent experiments (*n* = 20–28 cells). *** *p* < 0.001 (Student’s *t*-test).

Autophagy depends on the cytoskeleton network [38]. We investigated the influence of diclofenac on phagophore formation, the initial step of autophagy. Phagophores appear at the endoplasmic reticulum or other membrane as class III phosphoinositide 3-kinase (Vps34) is activated by means of a complex including Beclin-1, Atg14, and Vps15 [43]. Phosphatidylinositol (3) phosphate (PtdIns(3)P) is produced on the phagophore by activated Vps34. We monitored phagophore formation using a PtdIns(3)P reporter, a WD repeat and FYVE domain-containing 3 (WDFY3) [27,44,45]. In nutrient-starved HepG2 cells (vehicle) expressing GFP-LC3 and mCherry-WDFY3, both autophagosomes (LC3 spots) and phagophores containing PtdIns(3)P (WDFY3-positive spots) increased (Figure 4A top row). SAR405, a Vps34 inhibitor [46], restrained the formation of autophagosomes and phagophores (Figure 4A bottom row). Diclofenac inhibits autophagosome formation and elicits compromised phagophore formation. Aggregated large phagophores were observed in diclofenac-treated cells (Figure 4A middle row). Quantification reveals that WDFY3-positive spot number was reduced but sum fluorescence intensity was not changed in diclofenac-treated cells compared with control cells (vehicle) (Figure 4B). This result demonstrates that diclofenac suppresses the appropriate distribution of phagophores in the microtubule network but has little effect on phagophore formation. Considering that formation and expansion of the omegasome [47], a ring-like initial extension on the endoplasmic reticulum, depends on actin polymerization [38], the inhibitory effect of diclofenac is specific for microtubule polymerization but not for actin polymerization. We reasoned that microtubule depolymerization by diclofenac results in the inhibition of microtubule-dependent phagophore movement followed by autophagosome formation.

### 3.5. Diclofenac Induces Fragmentation of Mitochondria and the Golgi during Cell Death

**Figure 5:**
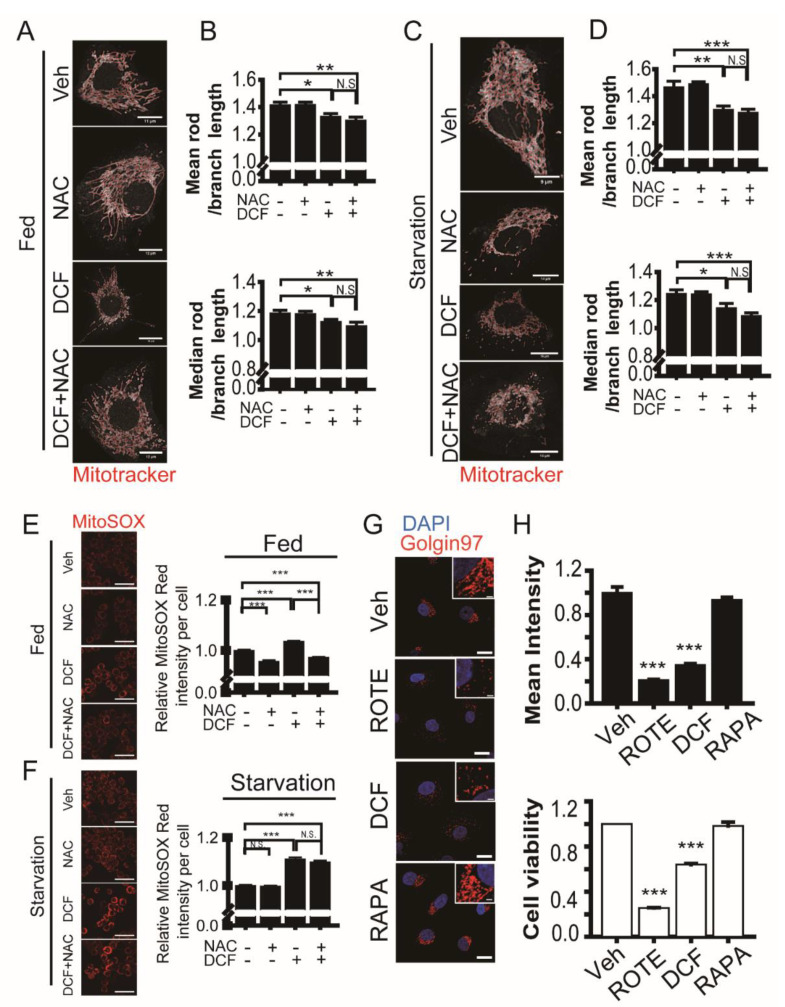
Diclofenac induces fragmentation of mitochondria and the Golgi during cell death. (**A**,**C**) Fed (**A,** Dulbecco’s modified eagle medium, 10% fetal bovine serum) or nutrient-starved (**C**, Earle’s balanced salt solution) HepG2 cells were incubated in a medium containing vehicle (0.1% dimethyl sulfoxide [DMSO]), *n*-acetylcysteine (2 mM), diclofenac (500 µM), or diclofenac (500 µM) with *n*-acetylcysteine (2 mM) for 8 h. Cells were stained with Mitotracker dye to measure mitochondria morphology. Images were obtained from confocal microscopy. Red line represents mitochondrial morphological skeleton for quantification. Scale bars are shown in each image. (**B**,**D**) Quantitative analysis for mitochondrial fragmentation. Ratio of mean rod length to branch length or that of median rod length to branch length of mitochondrial skeleton network per cell was calculated using the Mitochondrial Network Analysis toolset applied with Image J. Data are presented as means ± SD from three independent experiments (*n* = 9–13 cells). (**E**,**F**) Detection of mitochondrial reactive oxygen species using MitoSOX red. HepG2 cells were incubated in a medium containing vehicle (0.1% DMSO), rotenone (100 µM), rapamycin (0.25 µM), or diclofenac (500 µM) for 8 h. Cells were then imaged using a high-content imaging system (ImageXpress Confocal HT.ai) and red fluorescence mean intensity per cell was quantified. Data are presented as means ± SD from three independent experiments (*n* = 1589–2574 cells). Scale bar, 50 µm. (**G**) HepG2 cells were incubated in a medium containing vehicle (0.1% DMSO), rotenone (100 µM), rapamycin (0.25 µM), or diclofenac (500 µM) for 8 h. Cells were then subjected to immunofluorescence analysis with antibodies to Golgin97 (red). Nuclei were stained with 4–6-diamidino-2-phenylindole. Images were obtained from confocal microscopy. Scale bar, 20 µm; scale bar in inset, 2 µm. (**H**) Relative mean intensity of Golgin97 fluorescence in the perinuclear region of the cell is presented as means ± SD from three independent experiments (upper histogram, *n* = 42–50 cells). Cell viability assay using a water-soluble tetrazolium salt (EZ-cytox) is presented as means ± SD from three independent experiments (lower histogram). * *p* < 0.05; ** *p* < 0.01; *** *p* < 0.001 (Student’s *t*-test).

Fragmentation of mitochondria and the Golgi appears prior to irreversible cell death in cells under stresses such as increased ROS, increased cytosolic calcium ion, and endoplasmic reticulum stress [48,49]. Because Golgi fragmentation and mitochondrial dynamics are dependent on the microtubule network [50,51], we examined the effect of diclofenac as a microtubule destabilizer and autophagy inhibitor on mitochondria and Golgi morphology. Mitochondrial fragmentation in a single cell was quantified as a mean rod/branch length or a median rod/branch length using the ImageJ MiNA toolset [28]. Diclofenac caused mitochondrial network fragmentation in both nutrient-rich and nutrient-starved HepG2 cells (Figure 5A–D). The administration of *n*-acetylcysteine, a precursor of the antioxidant glutathione, into cells containing diclofenac did not alleviate mitochondrial fragmentation. We measured mitochondrial ROS levels using MitoSOX. Diclofenac induced mitochondrial ROS accumulation in HepG2 cells (Figure 5E,F). The treatment of *n*-acetylcysteine results in a decrease in mitochondrial ROS levels in nutrient-rich cells but not in nutrient-starved cells. In nutrient-starved cells, diclofenac further increased mitochondrial ROS accumulation by inhibiting autophagy flux. Administration of *n*-acetylcysteine did not neutralize the effect of diclofenac.

The Golgi is typically found as a stacked ribbon in the perinuclear region of the cell but reorganizes to peripheral sites under certain conditions including mitosis [52]. Inhibition of the activator for ADP-ribosylation factors leads to disassembly of Golgi by releasing many Golgi peripheral-membrane proteins [53]. Golgi fragmentation arises irreversibly by activated caspases during apoptosis [54]. Golgi fragmentation also occurs as an early pathological event prior to apoptosis in neurodegenerative diseases [55]. We measured Golgi fragmentation using Golgin97 fluorescence intensity in the perinuclear region of HepG2 cells treated with diclofenac, rapamycin, rotenone (an inducer of mitochondrial ROS production), and control cells (0.1% DMSO; vehicle). The Golgi was well-organized in the perinuclear region of control cells and rapamycin (a potent inducer of autophagy)-treated cells (Figure 5G,H). Rotenone and diclofenac treatment resulted in Golgi fragmentation and the collapsed Golgi was dispersed in the cytosol. This result is consistent with that of the cell viability assay using tetrazolium salt (compare upper and lower histograms in Figure 5H). Taken together with the result of mitochondrial fragmentation, the data show Golgi fragmentation can be an indicator of early cell death. Diclofenac induces mitochondria and Golgi fragmentation by destabilizing the microtubule network. This effect of diclofenac precedes an increase in H_2_O_2_ level. Superoxide anions, which are produced by mitochondria, mediate autophagy in nutrient-starved cells [56], and those produced by damaged mitochondria can be removed by superoxide dismutase 1. Therefore, superoxide dismutase 1 can further increase the cytotoxic effect of diclofenac by reducing autophagy.

### 3.6. Diclofenac in Combination with 5-Fluorouracil Induces Synergistic Cytotoxicity on Cancer Cells

**Figure 6:**
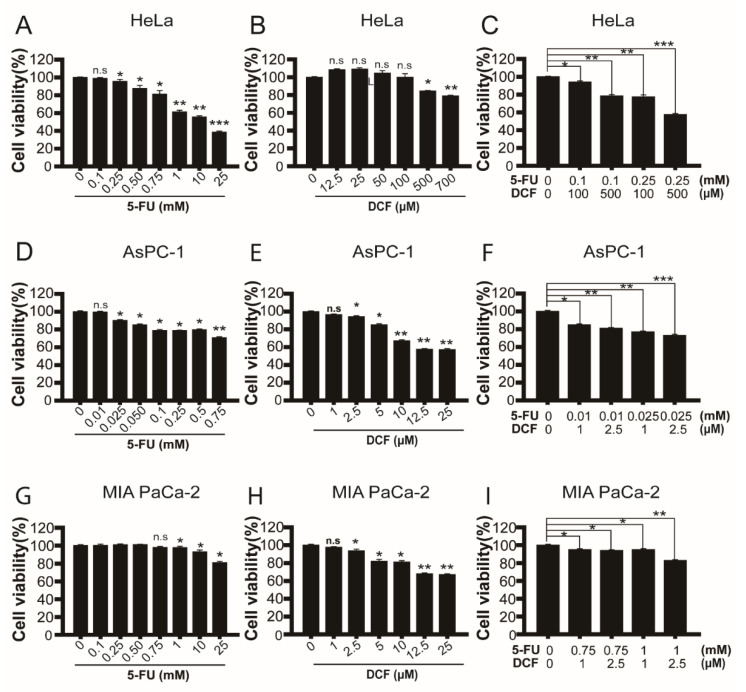
Cytotoxic effects of diclofenac alone or in combination with 5-fluorouracil on HeLa cells and two human pancreatic cancer cell lines (AsPc-1 and MIA PaCa-2 cells). (**A**–**I**) Cells were seeded into 96-well plates and treated with 5-fluorouracil and diclofenac alone or in combination, and cell survival was assessed 18 h after the treatment using EZ-cytox water-soluble tetrazolium salt assay. HeLa, AsPc-1, and MIA PaCa-2 cells were treated with 5-fluorouracil, diclofenac, or 5-fluorouracil plus diclofenac in combination at indicated concentrations where cytotoxicity or no cytotoxicity was observed. Combination treatment of 5-fluorouracil and diclofenac synergistically inhibited cell proliferation. Data are presented as means ± SD from three independent experiments. n.s, not significant; * *p* < 0.05; ** *p* < 0.01; *** *p* < 0.001 (Student’s *t*-test).

Drugs affecting microtubule polymerization have anticancer potential by inhibiting cell cycle progression and inducing cell death [57,58]. A representative agent is taxol, which increases microtubule stabilization, passes the mitotic spindle assembly checkpoint, and produces chromosomal instability followed by cell death [59,60]. We examined whether diclofenac induces cancer cell death more efficiently in combination with 5-fluorouracil than either drug does alone in HeLa cells and two pancreatic cancer cell lines (AsPc-1 and MIA PaCa-2) (Figure 6). The synergistic cytotoxicity of diclofenac with 5-fluorouracil was observed in all three types of cancer cells. In HeLa cells, treatment with 100 µM of diclofenac and 100 µM of 5-fluorouracil in combination showed cytotoxicity, whereas each treatment alone does not induce cell death. Similar synergistic cytotoxicity was observed in the two pancreatic cancer cell lines. Intriguingly, cytotoxicity of diclofenac to AsPc-1 and MIA PaCa-2 cells is more than 10 times greater than it is to HeLa cells. We reason that pancreatic cancer cells are highly sensitive to the treatment with diclofenac because these cells rely on upregulation of basal autophagy for their survival [61]. The results indicate that diclofenac can be a potent anticancer drug for certain types of cancers in combination with conventional anticancer medicines such as 5-fluorouracil.

## 4. Discussion

The IC_50_ of the NSAID diclofenac for Cox-2 is three times lower than that for Cox-1, indicating that diclofenac is a selective Cox-2 inhibitor [20]. Several studies have suggested that diclofenac has a preventive role against cancer. The inhibition of cell proliferation by diclofenac depends on reduced Cox-2 activity in chemical carcinogen-induced colon cancer in rats [62]. Although NSAIDs show the effects of cancer prevention by inhibiting inflammation via control of Cox activity, NSAIDs have also shown effects of cancer prevention independent of Cox activity [20,63]. However, the molecular mechanism by which a certain NSAID works for antineoplastic activity remains elusive and seems to differ according to cancer cell type and NSAID used.

**Figure 7:**
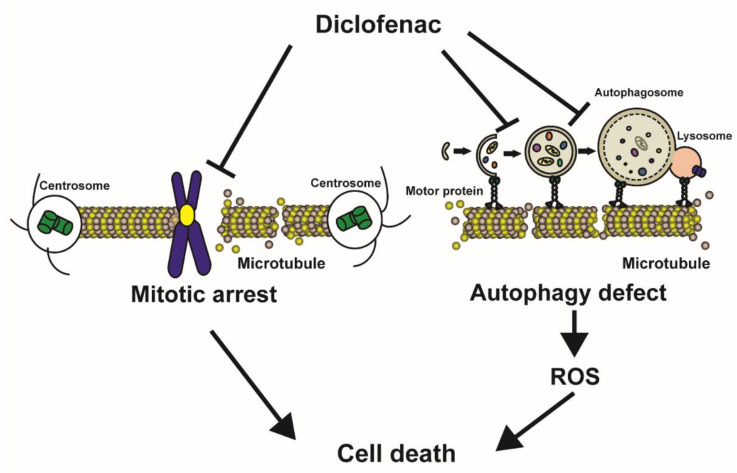
A model illustrating the death-inducing effect of diclofenac on cancer cells. Diclofenac induces microtubule depolymerization by direct binding. During the metaphase of mitosis, unattached kinetochores on chromosomes (caused by destabilization of mitotic spindles) initiate prolonged mitotic arrest followed by cell death. Diclofenac also inhibits microtubule-mediated phagophore mobilization and fusion of autophagosomes with lysosomes. Inhibited autophagy induces cell death through increased reactive oxygen species (ROS).

In this study, we propose a mode of action of diclofenac for specially induced cancer cell death (Figure 7). Diclofenac inhibits microtubule polymerization directly in vitro. Destabilized mitotic spindle filaments at metaphase elicit compromised spindle assembly checkpoints and interference with the formation of the spindle assembly checkpoint complex (Cdc20, Mad2, and BubR1), which is required to ensure proper mitotic progression [64]. Thus, cell death is induced by disturbing spindle assembly checkpoints, leading to chromosome mis-segregation, and aneuploidy, as has been reported in previous researches [65,66]. With respect to the regulation of autophagy, diclofenac appears to induce oxidative stress and lysosomal dysfunction [7]. We found that diclofenac specifically blocks the movement of phagophores and the fusion of autophagosomes and lysosomes due to microtubule destabilization. The inhibition of autophagy increases the accumulation of fragmented mitochondria and Golgi, and thereby produces cellular ROS from damaged mitochondria and gives rise to cell death. We observed that administration of the *n*-acetylcysteine antioxidant did not ameliorate mitochondrial fragmentation, indicating that inhibition of autophagy flux precedes increased ROS. We propose microtubules as cellular targets of diclofenac.

We demonstrated that diclofenac induces cancer cell death in combination with 5-fluorouracil. Several compounds disturbing the microtubule network result in anti-proliferation activity [51,58]. Most cancer cells display aneuploidy in their chromosomes while normal cells are euploid [67]. Aneuploidy is linked to genome instability, and further increasing genome instability has been a conventional and efficient strategy for cancer treatment. Both 5-fluorouracil and gemcitabine, two pyrimidine nucleoside antimetabolites, have been approved for the treatment of many cancers, including non-small cell lung cancer, pancreatic cancer, and breast cancer. They inhibit DNA replication and increase genome instability, ultimately inducing cell death [68,69]. Taxol, in combination with 5-fluorouracil or gemcitabine, results in cancer cell death synergistically through perturbation of the mitotic spindle assembly checkpoint followed by mitotic arrest [60]. Our data show that diclofenac in combination with 5-fluorouracil induces synergistic cell death in HeLa (cervical cancer), AsPc-1 (pancreatic cancer), and MIA PaCa-2 (pancreatic cancer) cells. Together with evidence that highly active autophagy is found in a variety of cancer types [70], our results showing that diclofenac inhibits autophagy through microtubule destabilization suggest the repositioning of diclofenac for combinational therapy with DNA replication-inhibiting drugs such as 5-fluorouracil and gemcitabine.

Long-term use of diclofenac can result in hepatotoxicity. For this reason, the appropriate in vivo concentration of diclofenac required to reach therapeutic effect on cancer tumors should be determined. Epidemiological studies show diclofenac has been implicated in liver injury from hepatotoxicity in the United States (53%; estimated incidence: 1–9 cases per 100,000 persons) [2,71]. Therapeutic doses of diclofenac for anti-inflammation are from 2 to 25 µM in human plasma [72]. In an overdose case with oral ingestion of 1500 mg of diclofenac, the plasma concentration reached 190 µM for 7 h [73]. The recommended dosage of diclofenac for the relief of osteoarthritis is 100 to 150 mg/day. When the maximum dose (150 mg/day) is allowed for oral ingestion, the plasma concentration seems to be 19 µM for 7 h and 5 µM for 24 h. Considering that diclofenac showed a half-maximal effective concentration of 170 μM for mitotic arrest and a half-maximal lethal dose of 200 µM for HeLa cells during 24 h-incubation, oral ingestion of 150 mg of diclofenac per day does not seem to be enough to induce anti-proliferation and death against cancer cells. However, the anticancer effect of diclofenac may vary according to the tumor type and location the body. In a systemic study [74] involving a pharmacokinetic analysis on oral diclofenac intake between 25 and 150 mg in humans, the time to reach maximal plasma concentrations is between 1.5 and 2 h after drug administration. Furthermore, the mean half-life in plasma concentrations is 1.2 h following a decline with a mono-exponential function. Diclofenac clearance takes between 3 and 4 h, consequently eliminating 90% of the drug. After an oral intake of 50 mg of diclofenac, the maximal diclofenac plasma concentration is 5.7 μM, and the clinically allowed maximum diclofenac dose for adult humans is 150 mg per day because of heart failure and chronic hepatic impairment. Given that the half-maximal lethal dose of diclofenac for HeLa cells is 200 µM, an oral intake of 50 mg of diclofenac is insufficient to achieve its antitumor activity.

We found that diclofenac is 100 times stronger in cytotoxic effect on AsPC-1 and MIA PaCa-2 pancreatic cancer cells than on HeLa cells (Figure 6). In combination with 5-fluorouracil, the minimum concentrations of diclofenac are from 1 to 100 µM for synergistic cell death, depending on cancer cell types. Autophagy was reported to be upregulated in pancreatic cancer [75,76], which is the one of most lethal cancers given that the 5-year survival rate of pancreatic cancer patients is about 10% in the United States [77]. Diclofenac can be a promising anticancer drug for cancers that show activated autophagy and deficiency of spindle assembly checkpoint. For combination therapy with 5-fluorouracil or gemcitabine, the effective concentration of diclofenac to kill in vivo cancer needs to be determined in the future in a variety of cancers while monitoring hepatotoxicity at the same time.

Structural stability and distribution of many cellular organelles depend on the microtubule network. Stress granules containing ribonucleoprotein and mRNA are generated by acute stress conditions, including oxidative stress. Microtubules control mobility and dynamics of stress granules [78]. Diclofenac induces chronic oxidative stress instead of acute stress and, for this reason, investigation into the relevance of diclofenac to control stress granule dynamics is of interest.

## 5. Conclusions

In this study, we demonstrate that microtubule destabilization by diclofenac, an NSAID, causes mitotic arrest and inhibition of phagophore movement and fusion of autophagosomes with lysosomes on the microtubule filament during autophagy. Diclofenac induces cancer cell death via compromised spindle assembly checkpoints and increased ROS. Diclofenac in combination with 5-fluorouracil, a DNA replication-inhibiting drug, has death-inducing effects on cancer cells. Pancreatic cancer cells, which have high basal autophagy, are particularly sensitive to cell death by diclofenac. These findings suggest the repositioning of diclofenac in combination with agents to block DNA replication for treatment of certain types of cancers.

## Data Availability

Data is contained within the article or supplementary material.

## Supplementary Materials

The following are available online at https://www.mdpi.com/article/10.3390/antiox11051009/s1, Figure S1: Uncropped western blots related with Figure 3E.
