# Supplementary figures and images for: Diclofenac: A Nonsteroidal Anti-Inflammatory Drug Inducing Cancer Cell Death by Inhibiting Microtubule Polymerization and Autophagy Flux
Source: Antioxidants (Basel). 2022 May 20;11(5):1009. doi: 10.3390/antiox11051009 (PMC9138099; doi:10.3390/antiox11051009)

**Figure S1. Uncropped western blots related with Figure 3E**

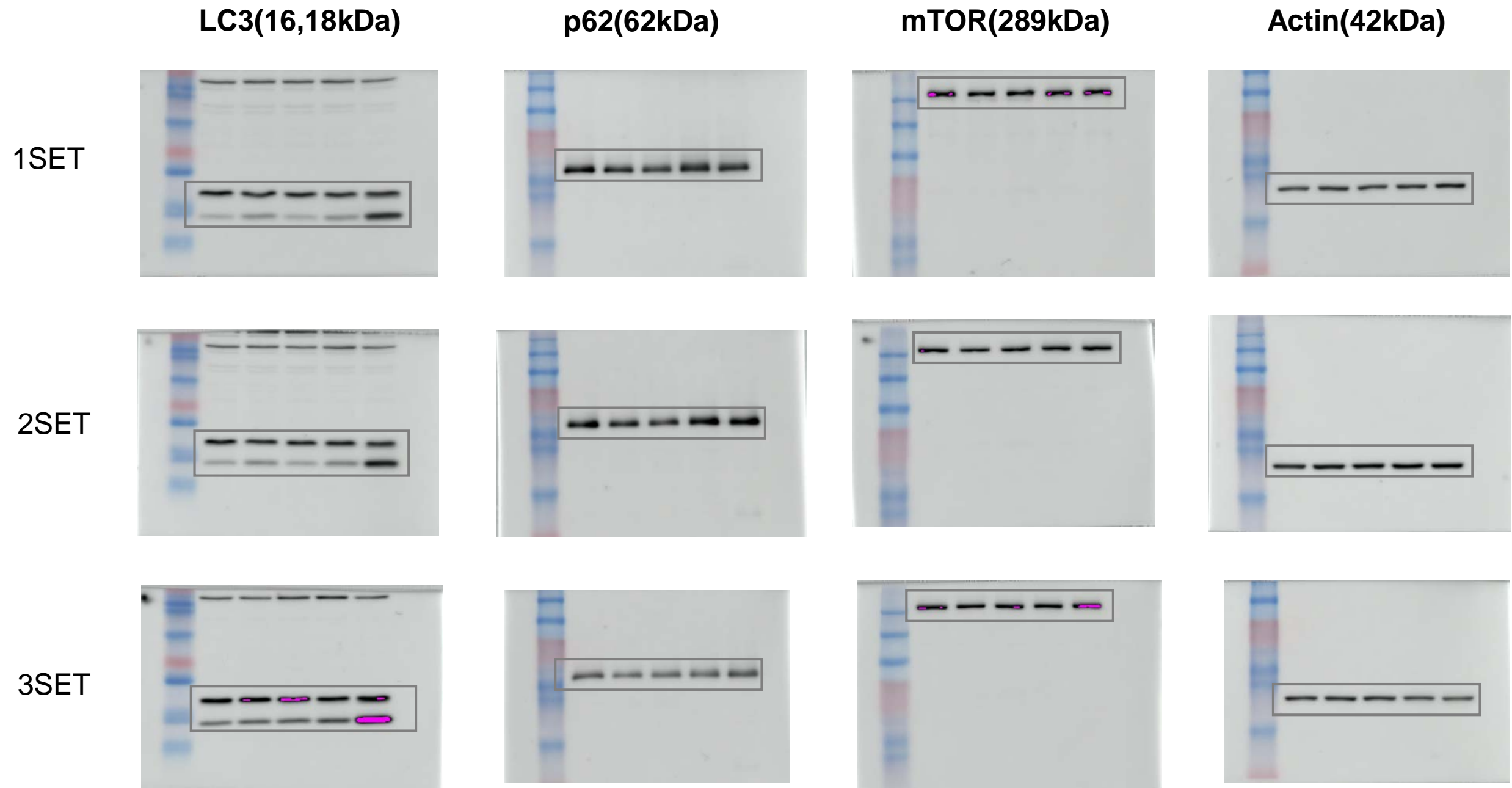

Supplement: Supplementary file 1 [file antioxidants-11-01009-s001.zip › antioxidants-1731343-supplementary.pdf]
